# Effect of home-based transcranial direct current stimulation (tDCS) on cognitive functioning in bipolar depression: an open-label, single-arm acceptability and feasibility study

**DOI:** 10.1186/s40345-025-00376-9

**Published:** 2025-03-17

**Authors:** Hakimeh Rezaei, Rachel D. Woodham, Ali-Reza Ghazi-Noori, Philipp Ritter, Elvira Bramon, Michael Bauer, Allan H. Young, Cynthia H. Y. Fu

**Affiliations:** 1 Department of Psychiatry and Psychotherapy, Faculty of Medicine, Technische Universität Dresden, Dresden, Germany; 2 Centre for Affective Disorders, Institute of Psychiatry, Psychology and Neuroscience, King’s College London, London, UK; 3 Department of Psychology, University of East London, London, UK; 4 Department of Psychiatry, University College London, London, UK; 5 National Institute for Health Research Biomedical Research Centre at South London and Maudsley NHS Foundation Trust, King’s College London, London, UK; 6 South London and Maudsley NHS Foundation Trust, Bethlem Royal Hospital, Beckenham, UK

**Keywords:** Transcranial direct current stimulation, Bipolar depression, Cognitive impairment, Home-based treatment, Neuropsychological tests, RAVLT, SDMT

## Abstract

Bipolar depression is commonly accompanied by cognitive impairments. Transcranial direct current stimulation (tDCS) is emerging as a novel non-invasive treatment for bipolar depression. Given the portability and safety of tDCS, we developed a home-based protocol with real-time supervision. Our aim was to assess the cognitive effects of a course of tDCS treatment in bipolar depression. 44 participants (31 women, mean age 47.27 years, SD 12.89) with bipolar depression of at least a moderate severity received 21 sessions of home-based tDCS over 6 weeks in an open-label design. The stimulation protocol involved 2 mA in a bilateral frontal montage (F3 anode, F4 cathode) for 30 min per session. Cognitive assessments were conducted at baseline and after the course of treatment: Rey Auditory Verbal Learning Test (RAVLT) to assess verbal learning and memory and Symbol Digit Modalities Test (SDMT) to assess psychomotor processing speed and visuospatial attention. 93.18% (n = 41) completed RAVLT and 59.09% of participants (n = 26) completed SDMT. A significant improvement was observed in RAVLT verbal learning score post-treatment (*p* = 0.002), which was not maintained following adjustment for improvement in depressive symptoms. In summary, a course of home-based tDCS in bipolar depression was associated with an improvement in verbal learning, which appeared to be related to improvement in depressive symptoms. These findings suggest potential benefits of tDCS for addressing cognitive impairments in bipolar depression, which can be investigated further in a sham-controlled design.

## Introduction

Cognitive impairments are a significant feature in bipolar disorder. Impairments are observed in all states of bipolar disorder, including in the euthymic phase (Minichino et al. 2015), and have detrimental effects on social and occupational functioning (Baune and Malhi 2015). Among these, impairments in verbal learning and memory, psychomotor speed, executive function, and sustained attention are most often observed (Keramatian et al. 2021).

Various treatment approaches have been investigated to improve cognitive functioning in bipolar disorder. Pharmacological interventions, for example lithium is initially associated with cognitive slowing effects in attention and memory, but may have fewer long-term negative effects, and in some cases, may help preserve cognitive function over time (Paterson and Parker 2017). Antipsychotic medications, such as quetiapine, can negatively impact on psychomotor speed, attention, and working memory (Sanches et al. 2015), while lurasidone has been associated with improvements in working and visual memory (Yatham et al. 2017). The mood-stabilizing medication, valproate, shows mixed effects on memory and executive function, while lamotrigine has been associated with improvements in verbal fluency and memory (MacQueen and Memedovich 2017; Sanches et al. 2015). Erythropoietin, a hormone with neuroprotective effects, has demonstrated some cognitive benefits, particularly in improving working and visual memory, although the long-term effects are less clear, and mifepristone, a glucocorticoid receptor antagonist, has shown positive effects on spatial working memory and verbal fluency (Watson et al. 2012; Young et al. 2004).

Non-pharmacological interventions include strategies aimed at addressing cognitive impairments. Cognitive remediation**,** which involves structured exercises to enhance attention, memory, and executive function, has shown potential in improving skills critical for daily activities (Keramatian et al. 2021; Solé et al. 2017). Functional remediation extends this approach by integrating cognitive training with real-world applications, enabling individuals to better manage the practical impact of cognitive difficulties on their daily lives and occupational performance (Martínez-Arán 2011; Torrent et al. 2013).

Non-invasive brain stimulation techniques include repetitive transcranial magnetic stimulation (rTMS) and transcranial direct current stimulation (tDCS), which have shown potential in improving executive function, memory, and attention by modulating neural activity (Keramatian et al. 2021). Short-term cognitive benefits have been observed with rTMS in randomized controlled trials, though the long-term impact is unclear (Myczkowski et al. 2018; Yang et al. 2019). Effectiveness of these techniques has been inconsistent, with outcomes varying due to differences in stimulation protocols, targeted brain areas, and treatment duration (Solé et al. 2017).

tDCS is a novel non-invasive brain stimulation technique that can modulate brain activity by delivering a weak current (1–2 mA) to the scalp via electrodes. Meta-analyses of randomized sham-controlled trials have reported that tDCS significantly reduces depressive symptoms in bipolar disorder (SMD = − 1.18, 95% CI − -1.66 to − 0.69) (Hsu et al. 2024) and demonstrates a high response rate compared to sham stimulation (Mutz et al. 2018). Depressive episodes in bipolar disorder (bipolar depression) are typically more common and longer-lasting than manic episodes (Belmaker and Bersudsky 2004) and have a greater impact on functional impairment than hypomanic/manic episodes (Rosa et al. 2010).

Cognitive effects of tDCS in bipolar disorder have been mixed. Cognitive improvements have been observed in domains, such as visuospatial memory and executive functioning (Bersani et al. 2017; Minichino et al. 2015), information processing speed (Bersani et al. 2017; McClintock et al. 2020) as well as visuospatial memory, selective attention, working memory, verbal learning and recall (McClintock et al. 2020). In each study, the anode was over the left dorsolateral prefrontal cortex (DLPFC) and cathode over right cerebellar (Bersani et al. 2017; Minichino et al. 2015) or right DLPFC (McClintock et al. 2020; Tortella et al. 2021), and treatment durations were 15 sessions over 3 weeks (Minichino et al. 2015; Bersani et al. 2017) or 20 sessions over 4 weeks (McClintock et al. 2020), in an open-label active treatment (Minichino et al. 2015) or double blind, sham-controlled design (Bersani et al. 2017; McClintock et al. 2020). Moreover, McClintock et al. (2020) observed no significant difference between high and low dose of tDCS and the cognitive improvements were independent of mood effects.

Conversely, no significant cognitive benefits of tDCS have been found in sustained attention (Martin et al. 2015), processing speed, memory, language, inhibitory control, attention, working memory, or executive function (Martin et al. 2015; Tortella et al. 2021) following sham-controlled trials of a single session of tDCS (Martin et al. 2015) or 12 sessions over 6 weeks (Tortella et al. 2021). In these studies, the anode was over the left DLPFC and cathode over right cerebellar (Martin et al. 2015) or right DLPFC (Tortella et al. 2021).

tDCS trials have generally been conducted in clinical or research settings, requiring multiple visits to a clinic or research center per week, which can create barriers to access (Woodham et al. 2022). As tDCS devices are portable and have demonstrated safety in clinical settings, we developed a home-based tDCS treatment protocol (Woodham et al. 2022, 2024). Our fully-remote, randomised controlled trial of home-based tDCS in unipolar depression (major depressive disorder) showed significant improvements in depressive symptoms, along with high safety and acceptability (Woodham et al. 2024).

We sought to investigate the effects of home-based tDCS treatment with remote supervision on cognitive functioning in bipolar depression. In the current study (Ghazi-Noori et al. 2024), verbal learning and memory were assessed using the Rey Auditory Verbal Learning Test (RAVLT) (Rey 1964), which evaluates how well an individual can learn, retain, and recall verbal information across multiple trials and time delays, and psychomotor processing speed and visuospatial attention were assessed using the Symbol Digit Modalities Test (SDMT) (Smith 1991). Validity and acceptability of web-based neuropsychological testing has been increasingly supported, demonstrating reliability across various populations, including healthy older adults (Cyr et al. 2021), clinical groups, such as Parkinson’s disease (Binoy et al. 2023), multiple sclerosis (Eilam-Stock et al. 2021), and bipolar disorder (Miskowiak et al. 2021). Positive correlations with in-person pen-and-paper tests, demonstrate validity and make them an increasingly popular choice in both research and clinical settings (Lynham et al. 2022).We aimed to investigate change in performance following tDCS treatment and to compare cognitive functioning to a healthy control group of participants.

## Materials and methods

### Study design and tDCS protocol

The study was conducted in accordance with the Code of Ethics of the World Medical Association (Declaration of Helsinki) and approved by the London Fulham Research Ethics Committee. Participants were recruited via online advertisements, referrals from general practitioners, psychiatrist and community mental health teams. After study details were explained and any questions were answered, informed written consent was obtained electronically. The study was an open-label, single-arm acceptability and feasibility trial of home-based tDCS for bipolar depression (ClinicalTrials.gov: NCT05436613 registered on 23 June 2022 https//www.clinicaltrials.gov/study/NCT05436613). Assessments and follow up visits were conducted remotely in real-time by Microsoft Teams videoconference. Participants were also able to attend visits in person, but no participant chose to attend in person.

**Fig. 1:**
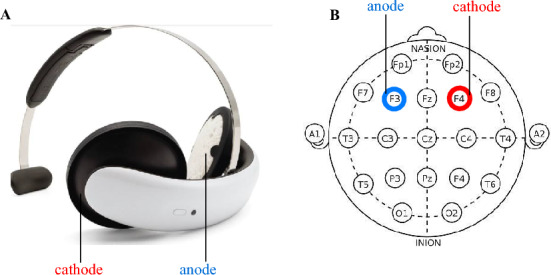
tDCS headset and electrode positioning diagram. **A** Figure depicting tDCS headset (Flow Neuroscience, Sweden). **B** The targeted stimulation locations at F3 and F4 are shown, with the anode in blue and the cathode in red, according to the International 10–20 EEG System

Following a comprehensive clinical assessment, the tDCS device (Fig. 1) was sent to the enrolled participant by post. A research team member would show each participant how to use the device in real-time by Microsoft Teams video conference.

The protocol consisted of 30-min active tDCS sessions in a bifrontal montage: the anode was placed at left DLPFC (F3 position according to the international 10/20 EEG system), and the cathode was placed at right DLPFC (F4 position). The stimulation was 2 mA for 30 min with a gradual ramp up over 120 s at the start and ramp down over 15 s at the end of each session. There were 5 sessions a week for 3 weeks, then 2 sessions a week for another 3 weeks, totalling 21 sessions. A minimum of 15 sessions was required for study completion. A member of the research team was present during each session providing a discreet presence with their camera on, while participants had both their camera and microphone enabled to facilitate communication if needed. Interaction between the participant and the team occurred only when the participant required assistance. During sessions, participants were allowed to read, use handheld devices, tablets, laptops, or desktop computers, or sit quietly.

Healthy controls were recruited through online ads or local outreach and completed baseline study activities and did not receive tDCS stimulation.

### Inclusion and exclusion criteria

Inclusion criteria for participants with bipolar depression: (1) adults aged 18 or older; (2) diagnosis of bipolar disorder, defined by Diagnostic Statistic Manual of Mental Disorders, Fifth Edition (DSM-5) (American Psychiatric Association 2013), in a structured clinical assessment using the Mini-International Neuropsychiatric Interview (MINI; Version 7.0.2) (Sheehan et al. 1998); (3) having at least a moderate severity of depressive symptoms as measured by a minimum score of 18 on Montgomery-Åsberg Depression Rating Scale (MADRS) (Montgomery and Åsberg 1979); (4) taking a stable dosage of mood-stabilizing medication for a minimum of two weeks or not taking any medication for a minimum of two weeks. Exclusion criteria included: (1) any concurrent psychiatric disorder, including obsessive compulsive disorder; (2) significant suicide risk, assessed by Suicidality module of the Mini-International Neuropsychiatric Interview (Sheehan et al. 1998), Montgomery-Åsberg Depression Rating Scale and 17-item Hamilton Depression Rating Scale (HDRS-17) (Hamilton 1960); (3) symptoms of mania or hypomania as measured by score greater than 8 on Young Mania Rating Scale (YMRS) (Young et al. 1978); (4) exclusion criteria for tDCS, including having a scalp or skin conditions, metallic implants; (5) history of epilepsy; (6) a history of seizures with loss of consciousness; (6) a history of neurological disorder or history of migraines.

Inclusion criteria for healthy control participants were adults at least 18 years old; and exclusion criteria were a personal or family history of psychiatric disorders, significant suicide risk, evidence of manic or hypomanic symptoms as measured by a Young Mania Rating Scale (Young et al. 1978) score greater than 8. Assessments were also conducted using the Mini-International Neuropsychiatric Interview (Sheehan et al. 1998).

### Clinical assessments

Clinical assessments for the bipolar depression group were conducted at baseline, week 2, week 6, with a follow-up assessment at month 5 (week 18) after the initial tDCS session. For the control group, assessments were conducted only at baseline. Assessments included the following scales: Montgomery-Åsberg Depression Rating Scale (MADRS) (Montgomery and Åsberg 1979), 17-item Hamilton Depression Rating Scale (HDRS-17) (Hamilton 1960), clinician-rated measure of depressive symptoms; Hamilton Anxiety Rating Scale (HAMA) (Hamilton 1959), clinician-rated measure of anxiety symptoms; Sheehan Disability Scale (SDS) (Sheehan 1983), self-report measure of disability and impairment; Patient Health Questionnaire-9 (PHQ-9) (Kroenke et al. 2001), self-report measure of depressive symptoms; Quality of Life Enjoyment and Satisfaction Questionnaire (Q-LES-Q) (Endicott 1993), self-report measure of quality of life; and Young Mania Rating Scale (YMRS) (Young et al. 1978), a clinician-rated measure of manic symptoms. Clinical response was defined as an improvement of 50% or greater in MADRS or HAMD scores from baseline. Clinical remission was defined as a MADRS score below 10 and HAMD score below 8. All ratings were completed by the same researcher under the supervision of principle investigator. Safety was assessed at each visit for any adverse events and in a formal questionnaire before and after each treatment session using the tDCS Adverse Events Questionnaire (Brunoni et al. 2011).

### Neuropsychological assessments

Neuropsychological assessments were conducted at baseline and after the 6-week treatment period. Alternative versions of the tests administrated in a counterbalanced order at each timepoint to control for repeated assessments and practice effects. IQ was measured using the Ammons Quick Test (Ammons and Ammons 1962). All assessments were conducted in real-time by videoconference.

The RAVLT was used to assess verbal learning and memory. The test involved two lists (A and B), each containing 15 words. The research team member first read list A aloud at one-second intervals, and participants were instructed to listen carefully and immediately recall as many words as possible after the reading was complete. The research team member recorded the correct responses. This procedure was repeated for five consecutive trials. After the fifth trial, List B, an interference list, was presented in the same manner. Following List B, participants were asked to recall as many words as they could from List A (Trial 6) without List A being reread. We calculated the following RAVLT scores: total learning (sum scores of Trials 1 to 5), which assessed the short-term verbal memory; learning over trials (subtracting five times the score of Trial 1 from the sum of the scores of Trials 1 to 5), measuring of verbal learning; and short-term percentage retention (total of Trial 6 divided by Trial 5, expressed as a percentage), assessing post-interference recall. A different version of the test was administered at the post-treatment session, following the same procedure.

The SDMT was used to evaluate information processing speed, psychomotor speed, and visuospatial attention. Two paper versions of the SDMT tests were mailed to the participants along with other study materials and clear instructions were provided during the baseline and final visit via video conference. The test included a grid displaying nine symbols paired with the numbers one to nine, which were shown below the symbols. On the same sheet, a larger grid displayed the symbols without corresponding numbers in the spaces below. Participants were asked to fill in the missing numbers in the empty spaces using the reference grid. They were instructed to use a pen or pencil to complete the task without skipping any symbols, starting as soon as the research team member began the timer and instructed them to start. The primary goal was to complete as many correct matches as possible within 90 s. The research team member closely monitored the participants during the task, ensuring accurate timing, and instructed them to stop when the time was up. Results were documented by capturing screenshots immediately after the task's completion. At the final session, an alternative version of the test was administered.

### Statistical analysis

Comparison of demographic variables between the bipolar depression and healthy control group consisted of independent sample t-tests for continuous variables (age, years of education and IQ), and Chi-square test for categorical variable (gender).

Repeated measures ANCOVA were conducted to evaluate the neuropsychological scores in bipolar depression group, with RAVLT (including AVLT total learning, AVLT learning over trials, and AVLT short-term percentage retention) and SDMT scores as the dependent variables and assessment time-point as the within-subjects factor, with two levels including baseline and end of treatment period. The analysis was performed twice: once with percentage change in depressive symptom severity as covariate and once without covariant adjustment. We used Pearson’s correlation to explore the association between MADRS percentage change and change in cognitive scores. To assess clinical scores, repeated-measures ANOVAS were performed with HDRS-17, MADRS, HAMA, YMRS, PHQ-9 and SDS score as the dependent variables and the assessment time-points as the within- subject factor, consisting of four levels: week 0, baseline (t_0_), week 2, after session 10 (t_1_), week 6, end of treatment period (t_2_). To assess the differences in neuropsychological features between bipolar depression and control participants, independent samples t-tests were conducted. Statistical analyses were conducted using IBM SPSS for Mac version 29.0. All analyses were two tailed and a significance value of *p* = 0.05 was set. Values are presented as mean ± standard deviation (SD) unless otherwise specified.

## Results

### Participants

A total of 44 participants with bipolar depression (31 women) were enrolled, with a mean age of 47.27 ± 12.94 (mean ± standard deviation (SD)) years and a mean duration of illness of 18.98 ± 12.47 years. The healthy control group consisted of 28 adults (17 women) with a mean age of 44.68 ± 14.45 years. The bipolar depression group had significantly higher MADRS scores at baseline (24.59 ± 2.64) compared to healthy control participants (0.75 ± 1.07, p < 0.001) (Table 1 Demographic and clinical data at baseline).

**Table 1:** Demographic and clinical data at baseline

	BD group	BD RAVLT completers	BD SDMT completers	HC group
Total number (Female)	44 (31)	41 (29)	26 (26)	28 (17)
Mean Age (years)	47.27 ± 12.9	47.93 ± 13.15	51.42 ± 10.38	44.68 ± 14.45
Age range (years)	24–76	24–76	30–76	21–72
Years of education	16.30 ± 2.46	16.37 ± 2.66	16.27 ± 292	16.89 ± 2.11
IQ	100.66 ± 9.3	101.29 ± 9.31	102.77 ± 9.38	103.39 ± 8.77
Clinical rating				
MADRS	24.59 ± 2.64	24.95 ± 3.11	25.08 ± 3.68	0.75 ± 1.07
HDRS-17	19.98 ± 2.62	20.24 ± 2.4	19.85 ± 2.49	0.82 ± 1.44
HAMA	16.55 ± 5.26	16.82 ± 5.23	16.07 ± 4.72	0.25 ± 0.51
PHQ-9	16.80 ± 4.94	16.98 ± 4.07	17.5 ± 4.15	1.36 ± 1.54
SDS	20.77 ± 5.87	20.83 ± 6.06	20.92 ± 6.5	0.46 ± 0.92
Duration of illness (years)	18.98 ± 12.47 Med:18.50	19.02 ± 11.33 Med:20.00	21.85 ± 11.4 Med:17.00	
Duration current depressive episode (weeks) (range)	49.55 ± 100.4 Med:20.00	50.68 ± 103.8 Med:20.00	63.62 ± 126.9 Med:20.50	
Previous number of episodes	18.16 ± 16.13	21.39 ± 24.13	24 ± 28.1	
Treatments during trial				
Taking mood stabilizer and other medications (%)	38 (86)	34(83)	19 (73)	
Taking antidepressant medication only (%)	1 (2)	1(2)	1(3)	
Taking no medication (%)	5 (11)	5(12)	5(20)	
Engaged in psychotherapy (%)	12 (27)	10 (24)	6 (23)	

Categorial variables are presented as number of participants with percentage in parentheses for treatment during trial. Mean values are presented with ± standard deviation

Med: median; BD: bipolar depression; HC: healthy control; RAVLT: Rey Auditory Verbal Learning Test; SDMT: Symbol Digit Modalities Test; MADRS: Montgomery-Åsberg Depression Rating Scale; HDRS-17: Hamilton Depression Rating Scale; HAMA: Hamilton Anxiety Rating Scale; YMRS: Young Mania Rating Scale; PHQ-9: Patient Health Questionnaire-9; SDS: Sheehan Disability Scale

41 participants (93.2%) (mean age 47.93 ± 13.15 years) completed the full 6-week course of treatment. 38 participants (86.3%) were taking mood-stabilizing medication, including Lamotrigine, Lithium, Quetiapine, Olanzapine, and Aripiprazole; 1 participant (2.3%) was taking antidepressant medication without mood-stabilizing medication, and 5 participants (11.4%) were not taking any pharmacological treatment. Additionally, 27.3% (n = 12) were engaged in psychotherapy (either CBT or psychodynamic psychotherapy) in combination with their medication. All 41 participants completed the RAVLT at week 6. To minimize potential delays and distractions associated with audio-based testing, we opted for the written version of the SDMT. However, this approach excluded participants who did not receive the printed test sheet in advance and, as a result could only perform the SDMT verbally. A total of 59.09% (n = 26) participants (mean age 51.42 ± 10.38 years) completed the written version of the SDMT at baseline and at week 6.

### Clinical assessments

At week 6 end of treatment, mean MADRS score was 8.91 ± 5.56 (F_(2,62)_ = 80.30, *p* < 0.001), with 34 participants (77.3%) showing a clinical response and 21 participants (47.7%) achieving clinical remission. Mean HDRS-17 score at week 6 was 6.77 ± 4.74 (F_(2,64)_ = 70.16, *p* < 0.001), with 37 participants (84.1%) demonstrating a clinical response and 31 participants (70.5%) achieving clinical remission. Significant improvements from baseline were also observed in HAMA, YMRS, PHQ-9, and SDS scores. Mean HAMA score at baseline was 16.6 ± 5.26 (range 9–36), indicating mild to moderate anxiety severity. After treatment, the mean score decreased to 6.36 ± 4.10 at week 6 and at follow up to 7.32 ± 4.85 at week 18, indicating mild anxiety. Mean YMRS score at baseline was 2.20 ± 1.49 (range 0–7), reflecting an overall absence of significant manic or hypomanic symptoms. This score further decreased to 0.80 ± 1.09 at week 6 and to 1.30 ± 1.37 at week 18 follow up, demonstrating a reduction in manic or hypomanic symptoms. Mean PHQ-9 score at baseline was 16.8 ± 4.02 and improved significantly after treatment, with a mean score of 6.52 ± 4.69 at week 6 and was maintained at week 18 follow up, with a mean score of 8.34 ± 5.68. SDS ratings of functional impairment were high at baseline, with a mean score of 20.77 ± 5.87, and showed significant improvement, with a mean score of 9.93 ± 7.85 at week 6 end of treatment and was maintained at follow up, with a mean score of 11.16 ± 8.87 (Ghazi-Noori et al. 2024).

### Neuropsychological assessments

At baseline, significant differences were observed in RAVLT total learning with healthy controls showing higher scores (51.50 ± 10.80) compared to the bipolar depression group (45.29 ± 11.00; *p* = 0.024). No significant group differences were found for RAVLT learning over trial (BD = 16.63 ± 6.31, HC = 16.32 ± 8.77; *p* = 0.864), RAVLT short-term percentage retention (BD = 83.16 ± 21.65, HC = 90.81 ± 11.00; *p* = 0.090), or the SDMT (BD = 44.35 ± 19.94, HC = 47.15 ± 8.49; *p* = 0.302) (Table 2).

**Table 2:** Neuropsychological test scores for bipolar depression participants and healthy control participants at baseline

	BD	HC	F-value	P-value
RAVLT total learning	45.29 ± 11.00	51.50 ± 10.80	0.074	0.024
RAVLT learning over trials	16.63 ± 6.31	16.32 ± 8.77	0.935	0.864
RAVLT short-term percentage retention	83.16 ± 21.65	90.81 ± 11	5.050	0.090
SDMT	44.35 ± 19.94	47.15 ± 8.49	0.305	0.302

Mean values are presented with ± standard deviation. RAVLT (n = 41), SDMT (n = 26), HC (n = 28)

BD: bipolar depression; HC: healthy control; RAVLT: Rey Auditory Verbal Learning Test; SDMT: Symbol Digit Modalities Test

#### Baseline and post-treatment effects

**Fig. 2:**
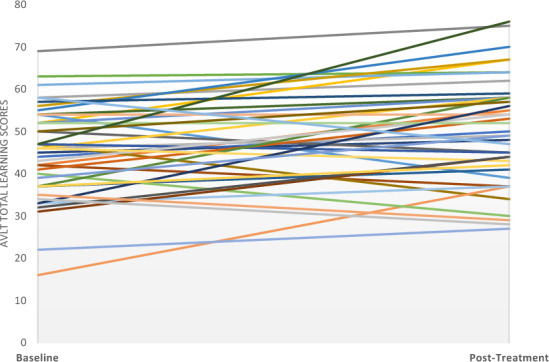
Change in short-term memory of verbal information over time. Shown are the estimated means of RAVLT Total Learning scores at baseline and after 6 weeks of tDCS treatment. Error bars represent 95% Confidence Intervals (CI) around the mean. RAVLT Total Learning scores range from 0 to 75, with higher values indicating better performance. A significant improvement was observed in the RAVLT Total Learning scores from 45.29 ± 11.00 at baseline to 50.29 ± 12.62 at week 6 (F _(1, 40_) = 10.672, p = 0.002)

In bipolar depression group, at week 6 end of treatment, significant improvements were observed in AVLT total learning scores, a measure of short-term verbal memory, with mean scores increasing from 45.29 ± 11.01 at baseline to 50.29 ± 12.62 post-treatment (F _(1,40)_ = 10.672, *p* = 0.002, η^2^ = 0.211) (Fig. 2). This change is notable, as it aligns with thresholds for reliable cognitive change, with improvements greater than 2.65 points on the AVLT sum of trials 1–5 scores, considered meaningful (Knight et al. 2007). No significant changes were found for AVLT learning over trials, a measure of verbal learning; (F_(1,40)_ = 0.475, *p* = 0.495; baseline: 16.63 ± 6.31; week 6: 17.73 ± 8.54), short-term percentage retention, a measure of post-interference recall (F_(1,40)_ = 0.108, p = 0.744; baseline: 83.16 ± 21.65; week 6: 84.46 ± 16.17) or in SDMT (F _(1,25)_ = 3.281, *p* = 0.082; baseline: 44.35 ± 19.94; week 6: 46.88 ± 11.01) (Table 3).

**Table 3:** Neuropsychological test scores for bipolar depression participants at baseline and end of treatment without covariate adjustment

	Baseline	Post-treatment	F-value	P-value
RAVLT total learning	45.29 ± 11.00	50.29 ± 12.62	10.672	0.002
RAVLT learning over trials	16.63 ± 6.31	17.73 ± 8.54	0.475	0.495
RAVLT short-term percentage retention	83.16 ± 21.65	84.46 ± 16.17	0.108	0.744
SDMT	44.35 ± 19.94	46.88 ± 11.01	3.281	0.082

Mean values are presented with ± standard deviation. RAVLT (n = 41), SDMT (n = 26)

BD: bipolar depression; RAVLT: Rey Auditory Verbal Learning Test; SDMT: Symbol Digit Modalities Test

After adjusting for depressive symptom severity by MADRS percentage changes, there were no significant effects in AVLT total learning (F_(1,39)_ = 0.000, *p* = 0.987), AVLT learning over trials (F_(1,39)_ = 0.007, *p* = 0.936), short-term percentage retention (F_(1,39)_ = 0.028, p = 0.867) or in SDMT (F_(1,24)_ 1.146, *p* = 0.295) (Table 4).

**Table 4:** Neuropsychological test scores for bipolar depression participants at baseline and end of treatment with covariate adjustment

	Baseline	Post-treatment	F-value	P-value
RAVLT total learning	45.29 ± 11.00	50.29 ± 12.62	0.000	0.987
RAVLT learning over trials	16.63 ± 6.31	17.73 ± 8.54	0.007	0.936
RAVLT short-term percentage retention	83.16 ± 21.65	84.46 ± 16.17	0.028	0.867
SDMT	44.35 ± 19.94	46.88 ± 11.01	1.146	0.295

Mean values are presented with ± standard deviation. RAVLT (n = 41), SDMT (n = 26)

BD: bipolar depression; RAVLT: Rey Auditory Verbal Learning Test; SDMT: Symbol Digit Modalities Test

No significant correlations were found between MADRS percentage change and changes in AVLT total learning (r = − 0.126, p = 0.431), AVLT learning over trials (r = − 0.056, p = 0.729), AVLT short-term percentage retention (r = 0.003, p = 0.985), or SDMT (r = − 0.271, p = 0.181).

### Adverse effects

The most common adverse effects were tingling (83.5%), skin redness (40.6%), itching (29.3%), and a burning sensation (26.5%). A total of 90.6% of adverse events related to tDCS were rated as mild, 9% as moderate, and 0.4% as severe. Severe adverse events included one report each of tingling and burning sensation, and two reports each of itching and skin redness (Ghazi-Noori et al. 2024).

## Discussion

Cognitive impairment in bipolar disorder affects daily functioning and quality of life, especially during depressive episodes (Cotrena et al. 2016). This study explored the impact of home-based tDCS with real-time remote supervision on cognitive functioning in BD in an open-label, single-arm acceptability and feasibility trial. Home-based tDCS was well tolerated, with mild, transient side effects (tingling, redness, itching) and no serious adverse events or mood switching (Ghazi-Noori et al. 2024). Improvements were observed in verbal learning, as measured by RAVLT total learning but not in verbal learning and post-interference recall following 6 weeks of tDCS treatment.

Participants with bipolar depression exhibited impairments in short-term verbal memory, as reflected by lower scores on RAVLT Total Learning compared to healthy controls (Malhi et al. 2007). However, no significant differences were observed between the groups in measures of verbal learning, post-interference recall or in psychomotor processing speed, as assessed by the SDMT. These findings may reflect the heterogeneity of cognitive impairments, particularly in verbal memory and processing speed, and the stability of cognitive performance in individuals with bipolar depression (Sparding et al. 2021).

Significant improvements were found in verbal learning, as measured by RAVLT total learning scores. However, when controlling for the percentage change in MADRS scores, these previously observed improvements became non-significant, suggesting that the improvement in RAVLT total learning scores may be influenced by changes in depressive symptom severity rather than a direct effect of the treatment. Deficits in verbal memory and processing speed in bipolar disorder have been observed during the euthymic phase, indicating that these impairments are trait-associated, rather than state-dependent, and can be independent of mood symptoms (Lee et al. 2014; Mann-Wrobel et al. 2011). Studies on the cognitive effects of tDCS in euthymic patients (Bersani et al. 2017; Minichino et al. 2015) have shown that three consecutive weeks of prefronto-cerebellar tDCS improve cognitive functions, particularly in visuospatial memory, executive functioning, and processing speed in euthymic bipolar disorder, suggesting that these cognitive impairments are likely independent of mood state changes. In unipolar depression, no significant improvements in cognitive functioning were observed despite improvements in depressive symptoms in our fully remote, randomized controlled trial of home-based tDCS (Woodham et al. 2024). The cognitive performance improvements observed in this study may be partially linked to changes in depressive symptom severity, but they may also occur independently of these changes.

The current findings support observations that tDCS can improve verbal learning and memory, selective visual attention, auditory attention, and information processing speed, and executive functioning in bipolar depression (McClintock et al. 2020), as well as in euthymic bipolar patients (Minichino et al. 2015; Bersani et al. 2017). In McClintock et al. (2020), repeated tDCS sessions were conducted over 4 weeks, with 20-min sessions applied 5 days per week in a sham-controlled, triple-masked design across six centers. The montage targeted the left DLPFC with the anode and right frontal area at F8 with cathode. Despite observed improvements in verbal learning, memory, selective attention, and processing speed, no significant differences were found between high and low stimulation dose. Minichino et al. (2015) applied tDCS over 3 weeks, with 5 sessions per week in an open-label study, targeting the left DLPFC with an anode and the right cerebellum with a cathode. Significant improvements were observed in visuospatial memory and executive functioning. Bersani et al. (2017) employed a 3-week, double-blind, sham-controlled design with 15 sessions (20 min each, 5 days per week), targeting the left DLPFC with the anode and the right cerebellum with the cathode. Active tDCS resulted in significant improvements in executive functioning, visuospatial memory, and attention compared to the sham condition. Improvements in SDMT performance have been observed in studies in both unipolar (Fregni et al. 2006) and bipolar depression (McClintock et al. 2020). However, some studies reported no significant improvements in RAVLT and SDMT after tDCS treatment compared to sham conditions in patients with major depressive disorder (Martin et al. 2018) and bipolar depression, despite initial SDMT improvements observed only in the latter (Loo et al. 2012). Fregni et al. (2006) used a 5-day protocol in unipolar depression and observed significant improvements in SDMT, while McClintock et al. (2020) applied a 4-week treatment in bipolar and unipolar depression, noting cognitive gains in SDMT and verbal learning but no significant differences between active and sham stimulation. In contrast, Martin et al. (2015), using a single tDCS session in euthymic bipolar patients, found no significant changes in cognitive outcomes. Tortella et al. (2021), with a 6-week protocol in bipolar depression, also reported no significant differences in cognitive outcomes, including RAVLT and other neuropsychological measures, between active and sham tDCS groups over six weeks. Loo et al. (2012) observed initial SDMT improvements in major depressive disorder following a single tDCS session, though these gains did not persist after 3 weeks of treatment. In all these studies, interventions were delivered in-person at clinical settings and the treatment durations ranged from 1 to 6 weeks. Nikolin et al. (2023) meta-analysis suggested that active tDCS effects continue with increasing treatment durations to 10 weeks. Significant tDCS effects were observed with 22 sessions over 10 weeks in the Brunoni et al. (2017) study, where active tDCS showed superiority to placebo in reducing depressive symptoms at the 10-week endpoint. In our home-based tDCS trial in major depressive disorder, a significant effect of active compared to sham tDCS was evident at the 10 week end of treatment (Woodham et al. 2024). It is possible that longer treatment durations could lead to better clinical outcomes in bipolar depression as well as beneficial effects on cognitive functioning.

In comparison to pharmacological treatments, tDCS has demonstrated more consistent cognitive improvements (Solé et al. 2017). Medications such as lithium and antipsychotics (e.g., quetiapine), often show mixed results. Some studies report attention and memory deficits within 14 days of lithium treatment, while others find no short-term impact. Quetiapine has been linked to impairments in psychomotor speed, attention, and working memory in one study, while another study found it to be less associated with verbal memory impairment compared to olanzapine or risperidone (Sanches et al. 2015). Tamura et al. (2021) evaluated the effects of erythropoietin, a neuroprotective hormone, in bipolar depression, finding improvements in working and verbal memory as measured by RAVLT. However, they also reported that these cognitive benefits diminished over time.

Improvements in cognitive functioning have a significant impact on daily functioning and quality of life, particularly during depressive episodes, where these deficits often contribute substantially to disability (Rosa et al. 2010). Our results demonstrated significant improvements in disability and functional outcomes, as measured by SDS and PHQ-9 scores, highlighting the potential of home-based tDCS to enhance real-world outcomes, including patients' ability to perform daily activities. These changes may represent early indications of potential functional benefits rather than broader, sustained improvements in functioning, which require long-term assessment.

Limitations of this study include the absence of a sham treatment arm, as all participants received active tDCS in an open-label design in which the observed effects on clinical and cognitive functioning could, in part, be attributed to a placebo effect. Additionally, the small sample size may have limited the power to detect subtle differences and draw definitive conclusions. The greater proportion of female participants and predominantly white ethnicity could limit generalization of the findings. The real-time presence of the researcher during each session may have contributed to improvements in depressive symptoms, as the experience of being observed and cared for can enhance their feeling of emotional security and overall well-being (Cruwys et al. 2014; Rimmer et al. 2024). As well, types of medications were not controlled. While participants were required to maintain a stable dosage of mood-stabilizing medication for at least two weeks or abstain from medication for the same duration, mood stabilizers such as lithium and lamotrigine exert their effects through the modulation of cortical excitability, which may influence tDCS efficacy (Lee et al. 2022). Most participants had been recruited through online advertisements, which may limit the generalizability of the findings to individuals less familiar with digital platforms. Finally, the lack of long-term assessment to determine whether the cognitive improvements observed following repeated tDCS sessions are sustained over time.

## Conclusion

A course of home-based tDCS in bipolar depression was associated with an improvement in verbal learning in bipolar depression. Since all participants had received active tDCS in an open-label design, it remains unclear whether this improvement resulted from the alleviation of depressive symptoms or if it reflects a direct effect of tDCS on cognitive functioning. These findings indicate the potential for home-based tDCS to improve cognitive functioning in bipolar depression. Further investigation in studies with larger sample sizes in randomized controlled trials is required to assess effects on cognitive functioning as well as long-term effects.

## Data Availability

The anonymised datasets used and/or analysed during the current study are available from the corresponding author on reasonable request.

## Abbreviations

BD: Bipolar depression
DSM-5: Diagnostic Statistic Manual of Mental Disorders, Fifth Edition
DLPFC: Dorsolateral prefrontal cortex
HC: Healthy control
mA: milliAmpere
MADRS: Montgomery-Åsberg Depression Rating Scale
MDD: Major depressive disorder
Med: Median
MINI: Mini-International Neuropsychiatric Interview
RAVLT: Rey Auditory Verbal Learning Test
rTMS: Repetitive Transcranial Magnetic Stimulation
SDMT: Symbol Digit Modalities Test
tDCS: Transcranial direct current stimulation
